# Independent Monte Carlo dose calculation identifies single isocenter multi‐target radiosurgery targets most likely to fail pre‐treatment measurement

**DOI:** 10.1002/acm2.14290

**Published:** 2024-01-30

**Authors:** Brett Erickson, Yunfeng Cui, Markus Alber, Chunhao Wang, Fang Fang Yin, John Kirkpatrick, Justus Adamson

**Affiliations:** ^1^ Department of Radiation Oncology Duke University Medical Center Durham North Carolina USA; ^2^ Scientific RT Munich Germany

**Keywords:** dosimetric robustness, quality assurance, single isocenter multi‐target radiosurgery

## Abstract

**Purpose:**

For individual targets of single isocenter multi‐target (SIMT) Stereotactic radiosurgery (SRS), we assess dose difference between the treatment planning system (TPS) and independent Monte Carlo (MC), and demonstrate persistence into the pre‐treatment Quality Assurance (QA) measurement.

**Methods:**

Treatment plans from 31 SIMT SRS patients were recalculated in a series of scenarios designed to investigate sources of discrepancy between TPS and independent MC. Targets with > 5% discrepancy in DMean[Gy] after progressing through all scenarios were measured with SRS MapCHECK. A matched pair analysis was performed comparing SRS MapCHECK results for these targets with matched targets having similar characteristics (volume & distance from isocenter) but no such MC dose discrepancy.

**Results:**

Of 217 targets analyzed, individual target mean dose (DMean[Gy]) fell outside a 5% threshold for 28 and 24 targets before and after removing tissue heterogeneity effects, respectively, while only 5 exceeded the threshold after removing effect of patient geometry (via calculation on StereoPHAN geometry). Significant factors affecting agreement between the TPS and MC included target distance from isocenter (0.83% decrease in DMean[Gy] per 2 cm), volume (0.15% increase per cc), and degree of plan modulation (0.37% increase per 0.01 increase in modulation complexity score). SRS MapCHECK measurement had better agreement with MC than with TPS (2%/1 mm / 10% threshold gamma pass rate (GPR) = 99.4 ± 1.9% vs. 93.1 ± 13.9%, respectively). In the matched pair analysis, targets exceeding 5% for MC versus TPS also had larger discrepancies between TPS and measurement with no GPR (2%/1 mm / 10% threshold) exceeding 90% (71.5% ± 16.1%); whereas GPR was high for matched targets with no such MC versus TPS difference (96.5% ± 3.3%, *p* = 0.01).

**Conclusions:**

Independent MC complements pre‐treatment QA measurement for SIMT SRS by identifying problematic individual targets prior to pre‐treatment measurement, thus enabling plan modifications earlier in the planning process and guiding selection of targets for pre‐treatment QA measurement.

## 1 INTRODUCTION

Stereotactic radiosurgery (SRS) can be used to treat patients presenting with benign, malignant, and/or functional disease in the brain.^1^, ^2^, ^3^, ^4^, ^5^ In addition, SRS delivered using a volumetric modulated arc therapy (VMAT) technique has been shown to be an effective and efficient method to treat multiple targets scattered throughout the brain with a single isocenter.^6^, ^7^, ^8^ Yet this technique presents challenges for pre‐treatment physics quality assurance (QA) as multiple scattered targets throughout the brain must be verified simultaneously in a setting of high dose gradients and lack of electronic equilibrium.

Several commercial measurement‐based solutions offer high spatial resolution and allow for true composite SRS plan measurements, such as the SRS MapCHECK (Sun Nuclear Corporation, Melbourne, FL, USA),^9^ myQA SRS (IBA, Louvain‐La Neuve, Belgium),^10^ Octavius 4D (PTW Freiburg, Germany),^11^ and various film solutions.^12^, ^13^ These devices possess the necessary spatial resolution for SRS plan verification, but are often limited to sampling a single measurement plane, requiring a subset of targets to be selected for an efficient pre‐treatment patie–nt‐specific QA process.^14^

In addition to measurement‐based QA, which is able to detect errors related to plan deliverability, an independent dose calculation serves as another crucial component of pre‐treatment patient‐specific QA. Traditionally, independent dose calculations involved simple point dose calculations, but recent advancements have introduced sophisticated algorithms capable of volumetric calculations that properly account for electron transport.^15^ These algorithms can detect errors associated with the treatment planning system (TPS) and hold promise particularly for single isocenter multi‐target (SIMT) SRS^16^, ^17^, ^18^, ^19^ as they provide comprehensive 3D dosimetric and dose‐volume histogram‐based comparisons for each target being treated, thus adding an additional layer of robustness for SIMT SRS plan QA.

Despite the increasing utilization of sophisticated and volumetric independent dose calculations for QA purposes, there is limited published experience in employing Monte Carlo (MC)‐based independent dose verification specifically for SIMT SRS. While the American Association of Physicists in Medicine (AAPM) Task Group 219 (TG‐219)^15^ recently published a report on independent dose calculation for intensity modulated radiation therapy (IMRT), it did not focus on MC algorithms, nor provide specific recommendations for SIMT SRS. In a recent research effort, we commissioned and applied a commercial MC independent dose calculation algorithm for multi‐target SRS; the beam model was developed by the vendor with commissioning and validation measurements being carried out by the academic institution; the end result serving as a baseline for other clinics implementing the same algorithm for multi‐focal SRS.^19^ As there is need for further guidance regarding adoption of secondary MC dose calculations for SIMT SRS QA, the current work has two aims. First, we assess the magnitude and sources of individual target dose differences (DDs) between the TPS and independent MC dose calculation. Second, quantify to what extent these DDs persist in the pre‐treatment QA measurement.

## 2 MATERIALS AND METHODS

### 2.1 Patient cohort

Thirty‐one SIMT SRS patients previously treated at the Duke University Medical Center were used for this study. Ten of the plans (hereby referred to as Group 1) were used retrospectively after treatment delivery for the commissioning of the SciMoCa (SMC) software. The remaining 21 plans (hereby referred to as Group 2), were taken from SIMT SRS patients treated at our institution during a two‐month period after implementing the SMC software. Both groups were taken randomly from the general cohort of patients within the time period that they were treated, with the added selection criteria based on plan characteristics for Group 1 to ensure inclusion of a wide range in number of targets, distances from isocenter, and treatment volumes.

### 2.2 Treatment plans and dose calculation

All treatment plans utilized a TrueBeam STX linear accelerator with High Definition Multi‐Leaf Collimators (HDMLC) and a 6X Flattening Filter Free (6X‐FFF) beam. The TPS used to prepare all SIMT plans was the Eclipse External Beam TPS (Varian/Siemens Healthineers). Plans were calculated with the Anisotropic Analytical Algorithm (AAA, versions 13.6.23 or 15.6.03), which had been previously commissioned for use with SIMT SRS. Calculations were made using a 1 mm isotropic dose grid.

The independent dose calculation was performed using a MC calculation algorithm within the SMC software (version 1.7.1.5050, Radialogica LLC). The SMC beam model was customized specifically for SIMT SRS cases via an extensive commissioning process as reported in a previous publication.^19^ This commissioning process was much more extensive than a standard commissioning of an independent dose calculation algorithm, as this was the first implementation of the software in this type of treatment geometry (multiple small targets located off axis). The commissioning was carried out to match independent measured data (rather than to match the TPS), and included an extensive set of central axis and off axis output factors, profiles, PDDs, etc., as well as thorough end to end testing. The prior work serves as a baseline for clinics implementing the software with this technique, both in terms of implementing a beam model from this vendor for SIMT cases, and providing a benchmark for achievable accuracy to be expected. The SMC independent dose calculation takes RT Dose, RT Plan, RT Structure, and CT images as its inputs and was also carried out using a 1 mm isotropic dose grid. Dose to water was reported (rather than dose to medium). SMC allows for calculation with varying levels of statistical uncertainty (with the tradeoff being calculation time), labeled as “Fast”, “Fine”, and “Extra Fine”; for this study we used the “Fast” (hereafter referred to as “coarse” statistical resolution, 2% uncertainty) and “Extra Fine” (hereafter referred to as “fine” statistical resolution, 0.5% uncertainty) settings for calculations.

### 2.3 Aim 1: Agreement between TPS and SMC

**FIGURE 1:**
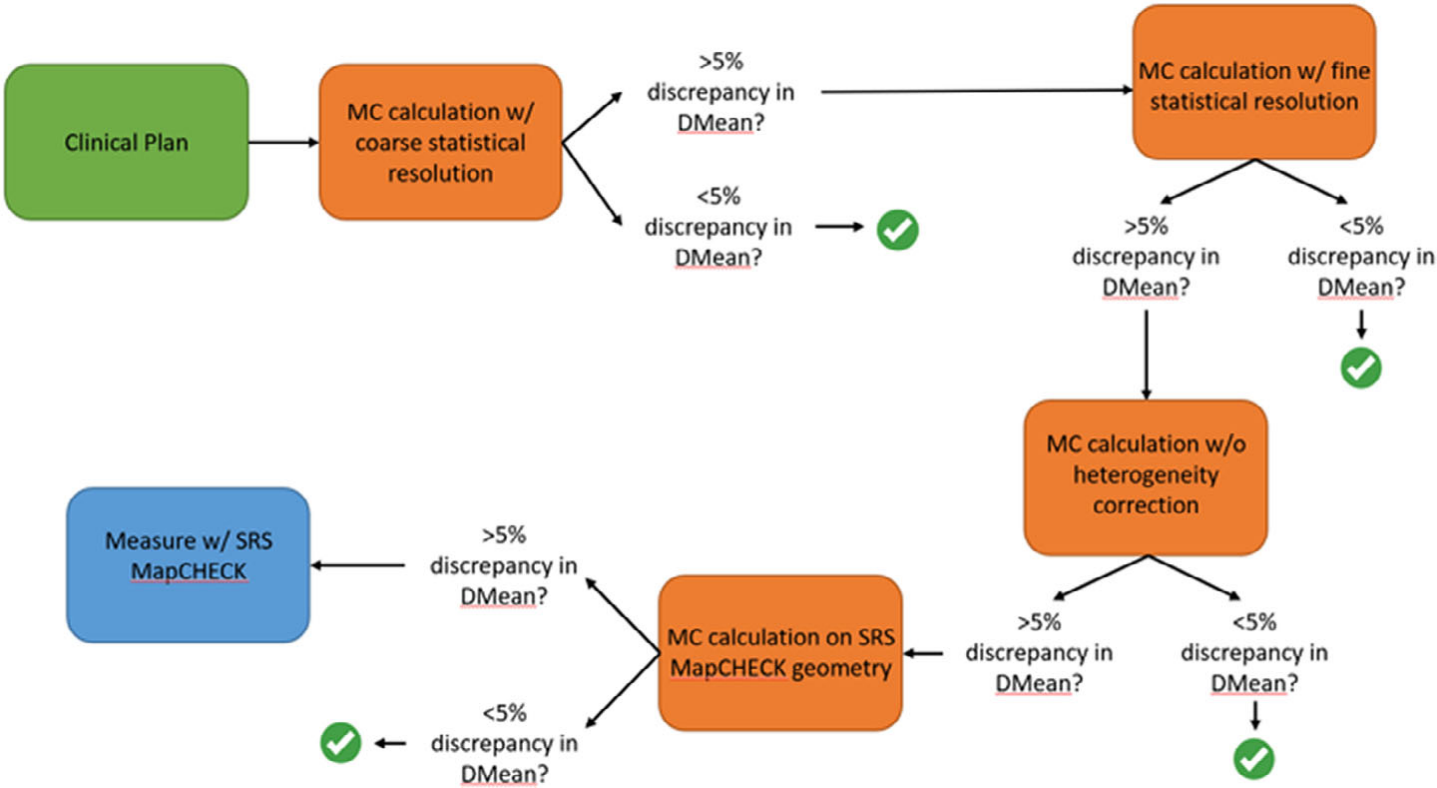
Workflow for identifying sources of disagreement between SMC and AAA. Evaluation was performed on a per‐target basis. Orange boxes represent calculations performed in SMC while blue boxes represent measurement. AAA, Anisotropic Analytical Algorithm; MC calculation w/, Monte Carlo calculation with; Measure w/, Measure with; SMC, SciMoCa.

Each of the SRS plans were recalculated with SMC in a sequence of scenarios designed to isolate sources of discrepancy between AAA and SMC. The progression is outlined in Figure 1 and is as follows:

1. SMC recalculation using coarse statistical resolution (fast calculation, 2% uncertainty)
2. SMC recalculation using fine statistical resolution (improved statistics, 0.5% uncertainty)
3. SMC recalculation without heterogeneity corrections (density override to 1 g/cc)
4. SMC recalculation in true composite geometry with SRS MapCHECK in StereoPHAN (model 1255, Sun Nuclear Corporation, Melbourne, FL, USA) geometry and target of interest centered on the detector plane

While minimal statistical uncertainty is desirable, fast calculations were initially performed to coincide with our clinical workflow. For dose calculations on SteroPHAN geometry, a density override to 1.19 g/cc was applied per manufacturer recommendations. Percentage differences were calculated between AAA and SMC on a per‐target basis for DMean[Gy], D99%[Gy], D95%[Gy], and D1%[Gy] (a negative value indicates SMC to be lower than AAA). The treatment plan was transferred to the StereoPHAN phantom and the isocenter moved so as to align the target of interest with the SRS MapCHECK detector plane. With this workflow, patient target contours are not transferred to the StereoPHAN CT dataset (due to no existing registration), and are not necessarily representative of the high dose volume on the StereoPHAN geometry. Thus, DVH metrics were collected and compared for a high dose volume defined as the volume encompassing 80% of the maximum dose delivered to a small sphere centered on the detector plane as determined by the AAA calculation. This isodose level was selected as it was found to have a volume comparable to the original target volume.

The number of targets exceeding differences of ± 5% and ± 7% for all metrics were tabulated at each test scenario, which correspond to the action criteria thresholds put forth in the TG‐219 report^15^ for independent dose calculations done in heterogeneous media in high dose/low gradient regions and low dose/high gradient regions, respectively. Both thresholds were investigated as SRS is a high dose/high gradient procedure. Note that only cases where any target was outside a ± 5% DD for DMean[Gy] moved on to the next step in the sequence, as outlined in Figure 1. As a part of SMC commissioning, cases from Group 1 proceeded through the entire workflow. Agreement within 5% for DMean[Gy] represents a “passing” QA result.

The magnitude of the DDs observed between AAA and SMC were investigated in regards to a variety of patient and plan parameters. Specifically, target distance from isocenter, target volume, MU‐weighted modulation complexity score^20^ (MCS, for whole plan, not per beam), and average equivalent multi‐leaf collimator (MLC) field size (for whole plan, not per beam) were collected for each target/plan. Each variable was used as an input in a multivariable linear regression model designed to investigate their effects on the magnitude of the dosimetric difference between AAA and SMC for DMean[Gy]. DMean[Gy] was selected for this analysis because it is currently the parameter of interest used in our independent dose calculations for clinical SIMT SRS cases. In order to increase the number of data points included in the regression analysis, the initial SMC calculations (coarse statistical resolution on patient anatomy and including tissue heterogeneity) were used as data input for the regression model.

### 2.4 Aim 2: Ability of SMC to accurately predict problematic SIMT SRS plans

Individual targets whose DD between TPS and SMC exceeded a ± 5% DMean[Gy] difference on StereoPHAN geometry were subsequently measured with the SRS MapCHECK embedded in the StereoPHAN (true composite delivery was used). Even though Group 1 had no cases exceeding a 5% difference on StereoPHAN geometry, all plans in Group 1 had at least one target measured (10 cases, 17 total measurements, 21 targets measured).^19^ Gamma pass rates (GPR) were calculated comparing the measured and calculated dose distributions, for both AAA and SMC. DD and distance to agreement (DTA) criteria of 2%/1 mm, 3%/1 mm, and 3%/2 mm were used with thresholds of 10%, 55%, and 80%. Further analysis of the high dose delivered to the detector (> 80% of DMax) was analyzed for each delivery.

To investigate how well SMC performance correlated with pre‐treatment measurement performance (i.e., does poor SMC performance result in poor measurement passing rates, and vice versa), targets that progressed all the way to the measurement phase (per Figure 1) and showed a persistent difference in DMean[Gy] between AAA and SMC were matched to targets with similar characteristics (i.e., similar target volume and distance from isocenter) but good agreement between AAA and SMC upon initial calculation. The matched targets were also measured with the SRS MapCHECK and GPRs (2%/1 mm and 10% threshold) were compared using a one‐sided *t*‐test.

**FIGURE 2:**
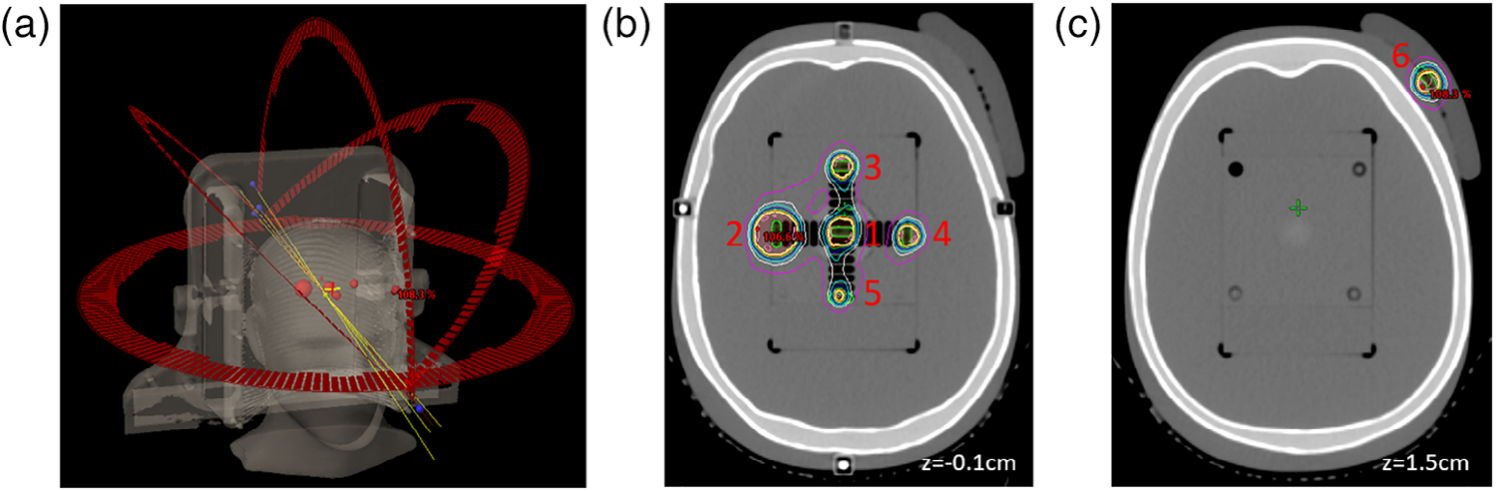
Images showing (a) beam geometry, (b) target spatial distribution at *z* = −0.1 cm, and (c) *z* = 1.5 cm. Target numbers are listed in red numbering next to the respective target. Identical geometries were used for Plans 1 and 2.

In addition, a similar analysis was also carried out using an anthropomorphic phantom geometry. Two SIMT SRS plans were optimized on an anthropomorphic stereotactic end‐to‐end verification (STEEV) phantom (CIRS Inc., Norfolk, VA, USA). Both plans shared the same beam and target geometry, with the plan consisting of six targets ranging in size from 0.05−4.13 cc and distance from isocenter ranging from 1.2−7.9 cm. The target and beam geometries can be seen in Figure 2. One plan (Plan 1) was optimized and showed good agreement between AAA and SMC (MCS = 0.0399) while the other (Plan 2) was over‐modulated (MCS = 0.0118) in order to induce a disagreement between AAA and SMC. Two conventional pre‐treatment measurements were carried out for both plans using the SRS MapCHECK in StereoPHAN. Measurement 1 was designed to sample as many targets as possible given the target spatial distribution within the phantom. This represents a clinically ideal scenario as it is desirable to verify as many targets as possible in a time sufficient for a busy clinic (i.e., a single measurement). Measurement 2 was designed to sample the target showing the largest disagreement between AAA and SMC in Plan 2. GPRs were compiled using 2%/1 mm, 3%/1 mm, and 3%/2 mm with a 10% low dose threshold.

## 3 RESULTS

### 3.1 Aim 1: Agreement between TPS and SMC

**FIGURE 3:**
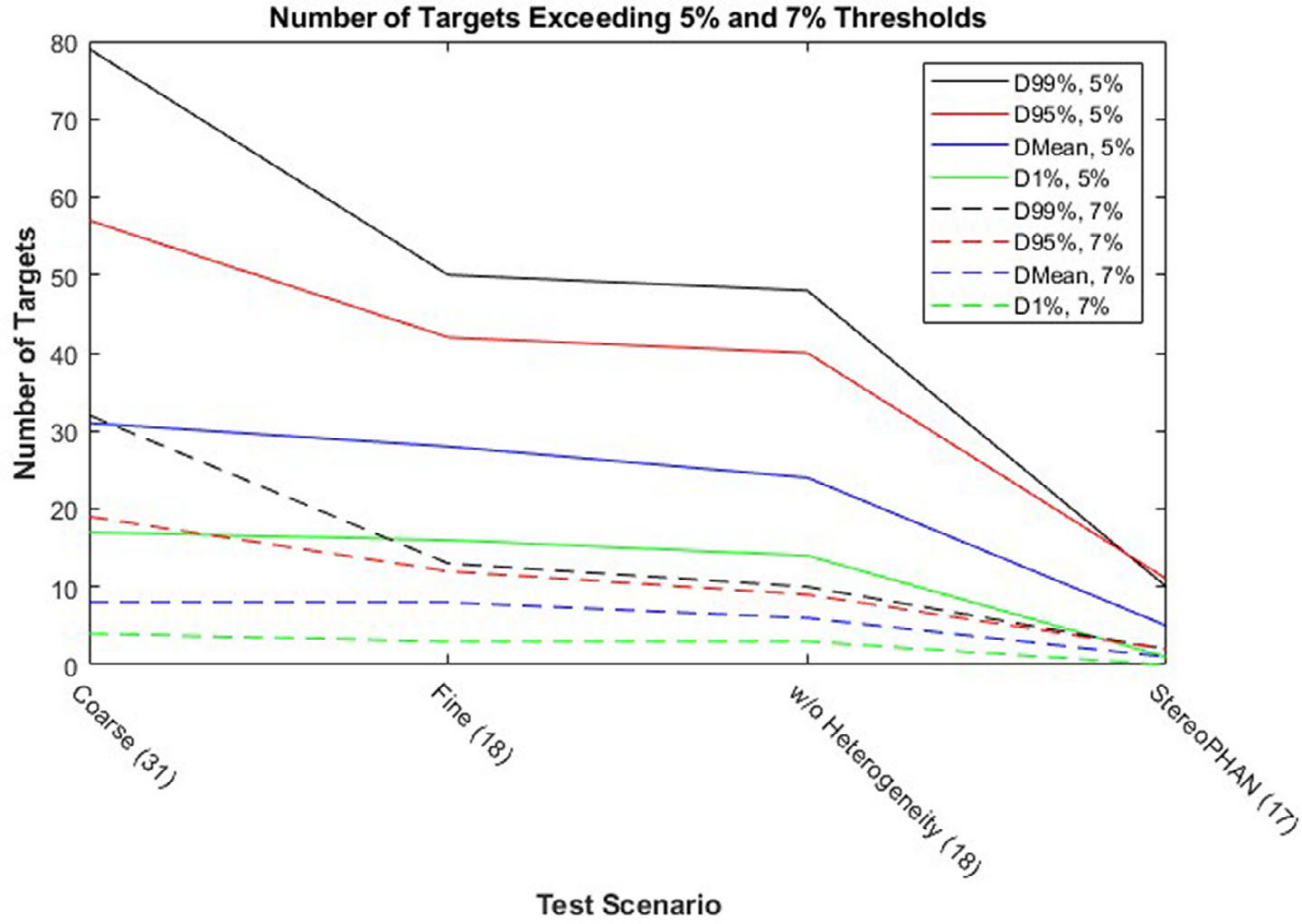
Number of targets exceeding 5% and 7% difference thresholds for each investigated dose metric at each successive test scenario. The number of plans calculated at each test scenario are listed in parentheses.

The number of targets per plan ranged from 2−20, totaling 217 individual data points among the 31 patients included in the study. The number of targets exceeding thresholds of 5% and 7% for each test scenario are illustrated in Figure 3. Based on Figure 3, 42% (*n* = 13) of all plans would have satisfied independent dose calculation upon initial calculation. In addition, it is apparent that the lower target doses investigated (D99%[Gy] and D95%[Gy]) were more influenced by statistical noise than the other two metrics (DMean[Gy] and D1%[Gy]), particularly D99%[Gy]. Removing heterogeneity only accounted for 14.3% (4/28) of targets which progressed to calculation without tissue heterogeneity. In contrast, calculation on phantom geometry accounted for 19/24 (79.2%) of targets that progressed to calculation on StereoPHAN geometry, indicating the difference in geometry between the patient and the phantom to be a potential source of discrepancy between AAA and SMC (due to varied depths along beam paths, etc.).

**FIGURE 4:**
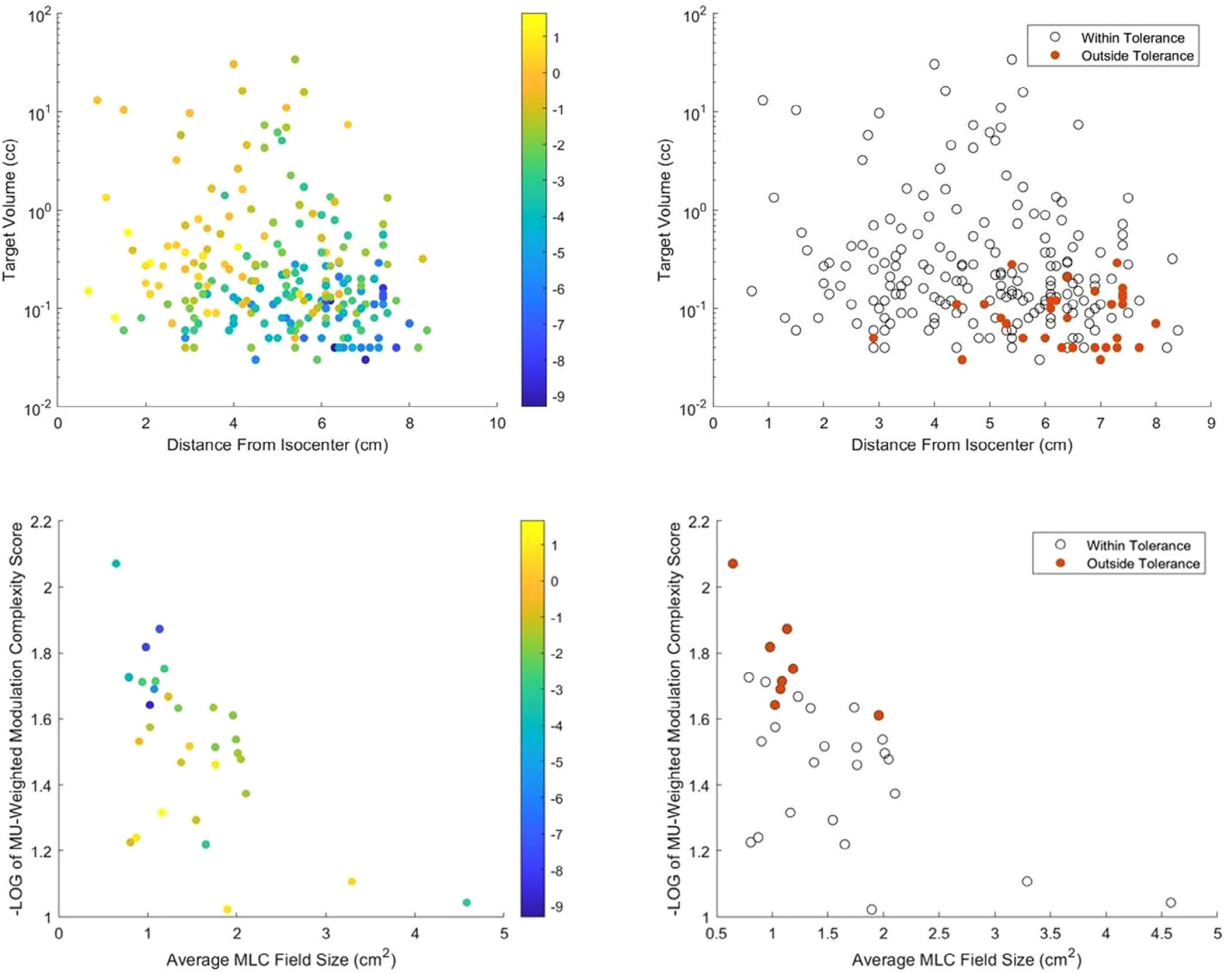
Color and binary plots showing the effects of target volume, distance from isocenter, MCS, and average MLC field size on the difference in DMean[Gy] between AAA and SMC. The color bar represents the percentage difference between AAA and SMC, with negative values indicating SMC to be lower. For the binary plots, red data points represent targets exceeding a 5% dose difference between AAA and SMC. AAA, Anisotropic Analytical Algorithm; MCS, modulation complexity score; MLC, multi‐leaf collimator; SMC, SciMoCa.

The ranges of target distance from isocenter, volume, plan average equivalent MLC field size, and plan MCS for the cohort were 0.7−8.4 cm, 0.03−34 cc, 0.65−4.58 cm^2^, and 0.0085−0.095, respectively. Color and binary plots showing the qualitative effect each of the aforementioned parameters has on the magnitude of the differences between AAA and SMC is shown in Figure 4. Generally speaking, larger discrepancies are observed between AAA and SMC for smaller targets positioned further from the isocenter, as well as for plans with smaller average MLC field sizes and a higher degree of plan modulation. To quantify this, the results of the multivariable regression are shown in Table 1. Larger distance from isocenter, smaller target volume, and increased plan modulation were all significant factors producing worse agreement between AAA and SMC. There was no statistically significant relation between the plan average MLC field size and the magnitude of the dosimetric difference.

**TABLE 1:** Effect of each of parameter on the percentage difference in DMean[Gy] between AAA and SMC.

Variable	Distance from Isocenter	Target Volume	MCS	Average MLC field size
Effect on difference in DMean[Gy]	−0.83% per 2 cm increase	0.15% per cc increase	0.37% per 0.01 increase	−0.29% per 0.5cm^2^ increase
*p*‐Value	**0.001**	**0.02**	**0.005**	0.2

*Note*: Negative values indicate that an increase in the variable causes SMC to be lower than AAA. Statistically significant *p*‐values are bolded.

Abbreviations: AAA, Anisotropic Analytical Algorithm; MCS, modulation complexity score; MLC, multi‐leaf collimator; SMC, SciMoCa.

### 3.2 Aim 2: Ability of SMC to accurately predict problematic SIMT SRS plans

Overall, there was good agreement between SMC, AAA, and the SRS MapCHECK measurements. The average GPR for all measured cases (per the workflow presented in Figure 1, includes cases from Groups 1 and 2) using different DD/DTA criteria and low dose thresholds are listed in Table 2. The average GPRs are relatively high when using a variety of DD/DTA/threshold combinations. Of note, the GPRs for SMC are noticeably higher than AAA when using a 10% dose threshold and 2%/1 mm criteria, indicating SMC to agree better with measurement than AAA. However, this separation seems to subside when using higher thresholds and looser DD/DTA criteria. Further analysis of the high dose region (>80% of DMax) delivered to the detector array reveals SMC to agree better with measurement compared to AAA, with average dose differences of 2.1% ± 2.1% and 4.9% ± 2.7%, respectively. When comparing AAA to measurement, it should be noted that 8/9 of the minimum GPRs observed came from plans belonging to Group 2 (9 total DD/DTA/threshold criteria were used). When comparing SMC to measurement, 6/9 of the minimum GPRs came from plans belonging to Group 2.

**TABLE 2:** GPRs comparing AAA/SMC dose to measurement.

DD/DTA	2%/1 mm			3%/1 mm			3%/2 mm		
Threshold	10%	55%	80%	10%	55%	80%	10%	55%	80%
AAA	93.1 ± 13.9 [45.0−100]	98.7 ± 2.6 [91.4−100]	97.3 ± 5.1 [85.0−100]	99.4 ± 1.6 [92.4−100]	99.3 ± 1.9 [94.3−100]	98.5 ± 4.1 [85.7−100]	99.9 ± 0.4 [98.0−100]	99.7 ± 1.2 [95.2−100]	99.3 ± 2.5 [88.9−100]
SMC	99.4 ± 1.9 [91.2−100]	97.6 ± 5.1 [78.6−100]	95.4 ± 9.3 [62.5−100]	99.7 ± 0.9 [95.6−100]	98.5 ± 3.9 [85.7−100]	97.1 ± 7.2 [75.0−100]	99.8 ± 0.8 [96.3−100]	99.3 ± 2.0 [92.9−100]	98.5 ± 4.4 [83.3−100]

*Note*: A variety of DD/DTA and threshold criteria were evaluated. Results are formatted as average ± standard deviation [range].

Abbreviations: AAA, Anisotropic Analytical Algorithm; DD, dose difference; DTA, distance to agreement; GPR, gamma pass rate; SMC, SciMoCa.

For the matched pair analysis, the targets progressing through the entire workflow in Figure 1 (problematic targets, all of which belong to Group 2) had average distances from isocenter and volumes of 6.7 ± 0.5 cm and 0.07 ± 0.05 cc, respectively, while the corresponding matched targets had average distances from isocenter and volumes of 6.7 ± 0.9 cm and 0.08 ± 0.04 cc, respectively. When comparing AAA to SRS MapCHECK measurement, the problematic targets showed much lower GPRs compared to their matched targets, with average GPRs of 71.5% ± 16.1% and 96.5% ± 3.3% (*p* = 0.01), respectively (2%/1 mm, 10% threshold). When comparing SMC to measurement, the problematic targets and their matched counterparts both showed average GPRs > 99% (*p* = 0.4), indicating no statistically significant difference between the two groups.

**FIGURE 5:**
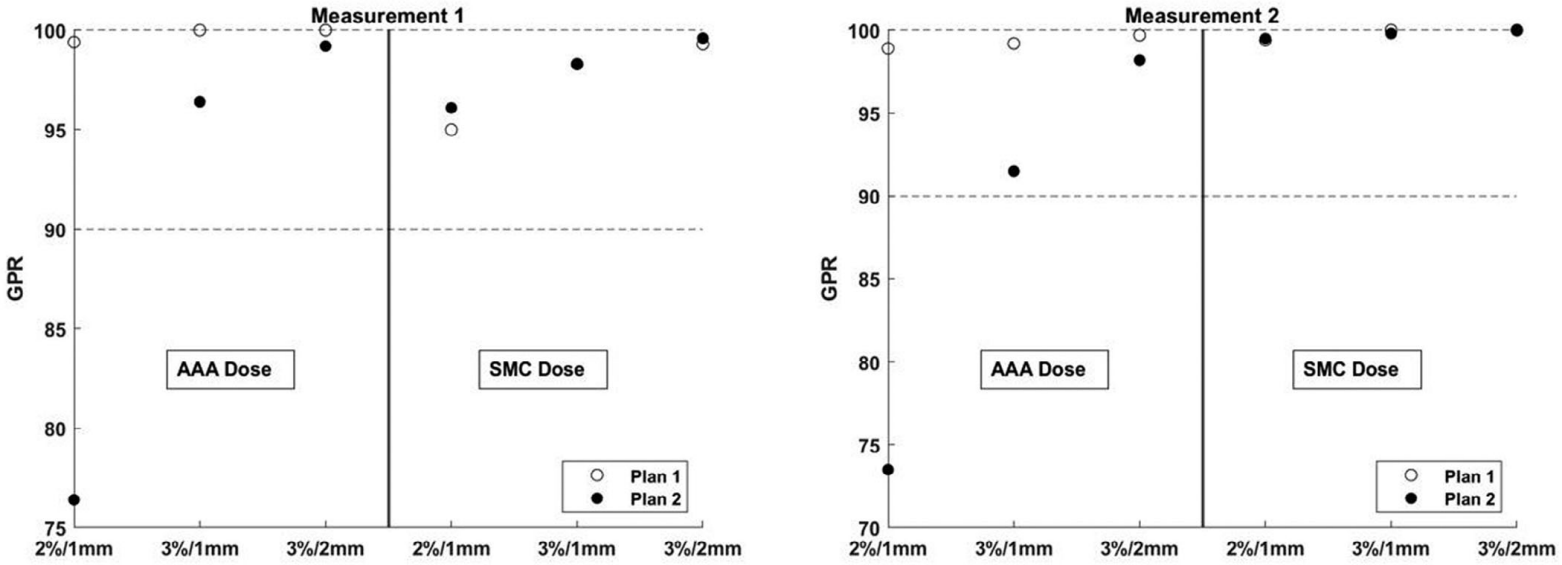
GPRs for Measurements 1 and 2 using the DD/DTA criteria specified on the *x*‐axis. The left half of each plot compares AAA to measurement while the right half compares SMC to measurement. A 10% dose threshold was used for all analyses. AAA, Anisotropic Analytical Algorithm; DD, dose difference; DTA, distance to agreement; GPR, gamma pass rate; SMC, SciMoCa.

For the STEEV treatment plans, Plan 1 showed excellent agreement between AAA and SMC for all six targets (difference in DMean[Gy] of 1.47% ± 1.08%, range 0.58%−3.47%) while Plan 2 showed worse agreement (difference in DMean[Gy] of 5.76% ± 2.56%, range 3.44%−10.6%). The target furthest from the isocenter (target 6 in Figure 2, distance from isocenter 7.9 cm, volume 0.48 cc) showed a difference in DMean[Gy] between AAA and SMC of 3.47% and 10.6% for Plans 1 and 2, respectively. Since this target showed the largest difference between AAA and SMC for Plan 2, it was selected as the target to be verified with conventional pre‐treatment measurement (Measurement 2). The other pre‐treatment measurement (Measurement 1) sampled targets 1, 2, and 4 as they lied in the same coronal plane (see Figure 2). GPRs for Measurements 1 and 2 using a variety of DD/DTA criteria and a 10% low dose threshold are illustrated in Figure 5. When comparing the AAA calculated dose to measurement, the highly modulated plan (Plan 2) had lower GPRs than Plan 1 for all DD/DTA combinations in both measurement scenarios. GPRs for Plan 2 fell below 90% when using 2%/1 mm, showing poor secondary dose calculation performance to correlate well with poor pre‐treatment measurement performance. When comparing SMC to measurement, GPRs for both plans were above 90% for all DD/DTA criteria and both measurement scenarios, showing an excellent correspondence between SMC and measured doses.

## 4 DISCUSSION

In this study we evaluated the agreement between the TPS and an independent MC software for SIMT SRS QA at our institution and explored its implementation as a QA tool. We found reasonable agreement between AAA and SMC for most of the targets treated as most agreed to within 5% for DMean[Gy]. In addition, we showed SMC to agree well with SRS MapCHECK measurements (all GPRs > 90% when using 2%/1 mm with a 10% threshold). The study by Lin and Ma^17^ reports results using an in‐house developed MC tool for three SIMT SRS cases (in addition to other sites). Their MC algorithm reports agreement with their TPS and measurement to be within 5% and 4%, respectively, for the SIMT SRS cases. The study by Ortega et al.^16^ shows excellent agreement between MC and measurement for SRS plans generated with HyperArc, with GPRs comparing TPS to MC > 97% using 2%/2 mm. Neither studies report DVH‐based comparisons, but both show MC performance to correlate well with TPS dose and measurement performance. These results are consistent with our study, and all show secondary MC to be an effective tool for SRS QA.

While it is apparent that MC can serve as a good tool for SIMT SRS QA, little literature exists regarding appropriate action criteria and effective integration with pre‐treatment measurement. The AAPM TG‐219 report recommends action criteria of 5%/7% for dose comparisons in heterogeneous media and high dose/low gradient regions, respectively. In a prior study, we applied the control process outlined in the AAPM TG‐218 report^21^ to DVH based criteria to determine custom action criteria for MC SIMT SRS calculations, and we found that a ∼7% action criteria was appropriate when comparing DMean[Gy] for individual targets.^19^ With this action criteria, 2.8% of targets (corresponding to 16.1% of plans) would not pass this action criteria even without heterogeneity correction, and 0.5% of targets (3.2% of plans) would not pass on the StereoPHAN geometry. The number of failures increases if a 5% action limit is used (2.3% of targets, 12.9% of plans failed on StereoPHAN geometry). The progression of test scenarios outlined in Figures 1 and 3 provide a useful workflow in troubleshooting dose differences when they arise. In addition, agreement between the TPS and independent MC correlates well with agreement in the pre‐treatment measurement when using strict DD/DTA criteria, both for targets showing good agreement and poor agreement. This has a number of practical benefits. First, we are able to actively identify problematic SIMT SRS plans before progressing to physician approval and subsequent physics QA tasks, thus potentially mitigating patient delays and adding an additional layer of plan robustness to SIMT SRS plan dosimetry. Second, the independent calculation can guide the selection of targets to be measured for pre‐treatment QA, thus improving the sensitivity of the pre‐treatment QA by focusing on targets that are most likely to have a discrepancy.

**FIGURE 6:**
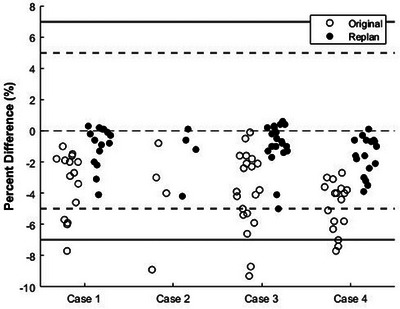
Distribution of per‐target difference in DMean[Gy] between AAA and SMC for the four re‐planned cases. Horizontal lines are drawn at ± 5%, ± 7%, and 0%. AAA, Anisotropic Analytical Algorithm; SMC, SciMoCa.

It is apparent in this work that not all targets treated in a single SIMT SRS plan will satisfy independent dose calculation, especially as the number of treated lesions increases. This raises the question of how to best handle this situation when it inevitably arises. To address this issue, we performed a follow‐up analysis in which all cases in this work which had one or more targets progress through the entire workflow presented in Figure 1 (*n* = 4 cases, five total targets) were re‐planned while maintaining similar plan quality to see if better agreement between AAA and SMC was attainable. Plan quality was evaluated based on various plan characteristics that were obtained from the TPS, including conformity index (CI), gradient measure (GM), MCS, and brain‐planning target volume (PTV) V60%[cc] (which corresponds to 12 Gy for a 20 Gy prescription). The CI is calculated as volume of the 100% isodose curve divided by the PTV volume, with conformity being greatest for CI = 1 and being less conformal for higher values of CI. GM is defined as the difference in cm between equivalent radii of the 50% and 100% isodose volumes; smaller values indicate a tighter falloff. MCS is a measure designed to quantify plan complexity, and is calculated using aperture area variability and leaf sequence variability over all control points of an arc as described elsewhere^20^; MCS ranges between 0 (extreme modulation) and 1 (no modulation). Our PTV expansion is 1 mm and brain‐PTV structure is the total PTV subtracted from the normal brain contour. For all cases, re‐planning was able to achieve agreement between AAA and SMC within 5% for all targets, as is shown in Figure 6. CI, GM, MCS, and brain‐PTV V60%[cc] changed by −0.1 ± 0.3 (−0.3−0.3), 0.13 ± 0.10 cm (0.0−0.20 cm), 0.012 ± 0.006 (0.004−0.019), and 2.1 ± 2.9 cc (−0.2−6.0 cc), respectively, between the original and new plans (positive values mean the re‐plan value is higher). The changes required to produce better agreement with secondary dose calculation were dependent on the case of interest. Some plans (3/4 re‐plans in this work) were sufficiently re‐planned by disabling jaw tracking and/or imposing a maximum MU constraint during optimization while others (1/4 re‐plans in this work) required a tweak to the collimator/arc geometry. While better agreement with secondary dose calculation was achievable, this analysis shows that re‐planning may result in slightly inferior (although comparable) plan quality when compared to the original plan, indicating there to be a potential interplay between plan quality and the robustness of the dose distribution. In addition, one must consider the time constraints imposed on the particular case, and hence we recommend to exercise clinical judgement when determining if re‐planning is necessary and how it should be performed.

It is important to recognize that while in this work the MC calculation consistently demonstrated good agreement with measurement, the agreement between the MC calculation and measurement observed should not be generalized to all cases. Instead, the independent calculation should be regarded as a valuable “second opinion” that can contribute to the overall assessment of treatment plan accuracy. When the independent calculation aligns with the primary dose calculation, a certain level of confidence may be instilled regarding the treatment plan's accuracy, while in situations when discrepancies between the primary and independent calculations arise it becomes necessary to conduct further investigation and exercise clinical judgment to determine the most appropriate course of action.

One of our findings was that the dose difference between the TPS and SMC was more likely to exceed the action criteria for targets that are smaller and farther from the isocenter. This agrees with the experience of other centers^14^ and agreed with measurement. From our analysis, at first glance it is surprising that the average MLC equivalent field size was not a significant parameter affecting agreement between AAA and SMC since uncertainties in small field dosimetry may be expected to increase these discrepancies. However, it is important to note that this parameter was acquired for the entire plan and may not be reflective of the average MLC field size surrounding a particular lesion. In addition, no information is contained in any of the investigated metrics regarding how close a particular lesion is to the field edge, which may be expected to increase dosimetric uncertainty due to uncertainties in penumbra modeling and can be exacerbated through the use of jaw tracking.

An unexpected result of this study can be seen in Figure 3. Removal of the patient geometry through calculation on StereoPHAN appears to remove some of the discrepancy between AAA and SMC. As a vast majority of these targets were not in the vicinity of the central axis, this reconciles nicely with the results of Table 1, namely that distance from isocenter is a significant factor affecting agreement between AAA and SMC. A conjectured explanation for these findings may lie in the uncertainties associated with the AAA beam model, specifically in regards to off‐axis small field output factors, small field off‐axis profiles and depth dose curves, MLC modeling, and penumbra modeling. Targets further off axis are more likely to be near the field edge, and hence may be directly subject to the uncertainties associated with penumbra modeling. While the aforementioned factors may contribute to uncertainties observed in the patient geometry, simpler phantom geometries may mitigate the cumulative effects of these factors and could also be a conjectured explanation for this observation. Another factor contributing to this observation may be that the actual target contours are not transferred to the StereoPHAN, and instead we used the DVH from the 80% isodose lines as a surrogate for the target due to it having similar volume to the original target volume.

Also, while it may be possible to improve the beam modeling of one or both calculation algorithms, it is worth noting that both algorithms underwent a rigorous commissioning and beam modeling process for SIMT SRS. Good agreement between the two algorithms was demonstrated for the majority of targets, while a dose difference manifested for only a small minority of cases, which could be mitigated via plan re‐optimization. In practice, it may always be possible to create a treatment plan which tests the limitations of a beam algorithm (via overmodulation, etc.). When the primary and independent calculations employ distinct algorithms and MLC modeling techniques (which is the ideal scenario for an independent calculation), plans that challenge the limits of one or both algorithms are likely to manifest as dose differences between the two calculations. Improvements in the Eclipse MLC model (such as the recently developed Enhanced Leaf Modeling in Eclipse v18) may help mitigate some of these differences,^22^ although further investigation remains warranted.

## 5 CONCLUSIONS

In this study, we found that discrepancies between the TPS and the MC calculation for individual targets of SIMT plans is influenced by target distance from the isocenter, target volume, and plan modulation, indicating the importance of considering these factors when evaluating SIMT SRS plans. These differences persisted in the pre‐treatment phantom measurements as shown by a matched pair analysis, and were successfully mitigated via plan re‐optimization with a focus on decreasing the plan modulation. Thus, the independent MC dose calculation can enhance SIMT SRS QA by enabling (1) comprehensive dosimetric feedback regarding all treatment targets, that is (2) predictive of the outcome from pre‐treatment QA, and is (3) provided at a sufficiently early stage in the planning process to allow for mitigation strategies to be employed.

## AUTHOR CONTRIBUTIONS

Author Erickson contributed to the design, data collection, and writing of the work. Author Cui contributed to the design and writing of the work. Author Alber contributed to the design, data collection, and writing of the work. Author Wang contributed to the design and writing of the work. Author Yin contributed to the design and writing of the work. Author Kirkpatrick contributed to the design and writing of the work. Author Adamson contributed to the design, data collection, and writing of the work. All authors approved the final version of this work.

## CONFLICT OF INTEREST STATEMENT

Drs Adamson and Kirkpatrick report ownership of Clearsight RT LLC, which is unrelated to this work. Dr Adamson reports research funding from Radialogica LLC, which is a distributor of the SciMoCa independent dose calculation software. Dr Alber is a founder of Scientific RT, which manufactures the SciMoCa Monte Carlo independent dose calculation algorithm.
